# Reconceptualising Person-Centered Service Models as Social Ecology Networks in Supporting Integrated Care

**DOI:** 10.5334/ijic.4222

**Published:** 2019-06-27

**Authors:** Geoff Woolcott, Robyn Keast, Peter Tsasis, Sebastian Lipina, Daniel Chamberlain

**Affiliations:** 1 School of Education, Southern Cross University, Lismore, AU; 2 School of Business and Tourism, Southern Cross University, Queensland, AU; 3 School of Health Policy and Management, York University, Toronto, CA; 4 Unidad de Neurobiología Aplicada (UNA, CEMIC-CONICET), Universidad Nacional de San Martin, Buenos Aires, AR; 5 The Australian Prevention Partnership Centre, School of Psychology and Public Health, La Trobe University, Victoria, AU

**Keywords:** social ecological networks, social network analysis, integrated care, person-centered approach

## Abstract

Efforts to address problems such as mental health, poverty, social exclusion, and chronic disease have often proven resistant to traditional policies or interventions. In this paper, we take up the challenge and present a pioneering new method of analysis in drawing on theoretical and methodological extensions of two prominent approaches, namely, social network analysis and developmental social ecology. Considered in combination, these two seemingly disparate approaches frame a powerful new way of thinking about person-centred care, as well as offer a methodologically more rigorous set of analytical tools. The conceptual model developed from this combination offers to bridge the apparent disconnect between service integration levels and patient needs in such a way as to direct optimal effort to interventions at the individual level and to provide a new innovative approach to the delivery of integrated care.

## Introduction

Universal services, such as health and education, as well as targeted or specialized interventions, have made an enormous contribution to overall societal wellbeing. There remains, however, a significant and increasing number of individuals with complex and multiple needs for whom such general services are not sufficient or who fall between the boundaries of the specialist silos [1]. Their needs arise from chronic diseases that have multiple medical and functional conditions which require a system of care that facilitates access to integrated community supports and extends across multiple providers and sectors [2,3].

Over the years, the increasing trend of populations with ongoing complex health and social needs is beginning to pose substantial challenges in healthcare expenditures [4]. Furthermore, a growing weight of international evidence is beginning to indicate that multi-morbidity is becoming the norm in many nations, rather than the exception [5]. As such, multi-morbidity is further raising challenges of integration for patients, healthcare providers, healthcare systems, and governments around the world [6,7]. While integrating care for populations promises better outcomes for people requiring multiple services, from multiple providers, integrating efforts have been slow with minimal success [8]. This is in part because integration challenges conventional professional boundaries, current norms of healthcare organizations, and delivery and practice. It also requires rethinking of the roles and responsibilities of healthcare professionals who are increasingly expected to work in intersectoral teams, engaging patients and informal caregivers as active participants and partners [9].

Network models that take local environments into account, including socio-economic conditions and resource priorities as well as local actors associated with different sectors such as health, education, and housing are emerging and evolving within context-bound sets of conditions [10,11]. With this in mind, this article focuses attention on a person-centered and place-based approach to delivering integrated care. We describe person-centered care as care that takes into consideration individual preferences and values in guiding all aspects of healthcare delivery [12]. Our thinking is framed by Ekman et al who emphasize personhood, equality, and customized outcomes, through paying attention to personal narratives, establishing partnerships and creating care plans [13]. We adopted Belefontaine’s and Wisener’s definition of a place-based approach as a collaborative process, where stakeholders engage to address issues as they are experienced within a geographical space, community, neighborhood, region, or eco-system [14]. We define integrated care as person-centered care that is coordinated and shared across professional and organizational settings and tailored to the client’s needs [15]. It is our understanding that integrated care requires that providers work together in partnership, and with the understanding of who should do what, with whom and why, and with the aim of meeting the client’s needs and preferences [16].

## Background

It is increasingly understood that interventions must do more than provide a service, they must take into account or explicitly consider the individual socio-cultural determinants, such as family supports, location and personal capacity, that can impact on a person’s ability to take-up and optimally engage with the services offered [17]. In fact, one of the greatest challenges has been identifying, assessing, and tracking formal and informal networks of support and including them into the care and treatment plan [18].

Ideally, linking inter-sectoral networks of professional and organizational activities across traditional boundaries (silos) can provide the context to individuals with complex needs, such as patients with co-morbidity, polypharmacy, frailty and mental health for the delivery of a personalized integrated approach to care [19]. By directing attention to the provision of a coordinated continuum of service and to the interrelationships of service providers, interventions can be effectively adapted to the individual’s functional status, cognition, co-morbidity, nutrition, medication, and social support needs [20]. Thus, this puts the right care in the right place, at the right time, for the right person.

There is now compelling evidence, from research across a number of fields of endeavour, highlighting the benefits of an authentic person-centred service approach; one that first identifies and takes into account the person’s needs, risks, strengths and context, and uses this as a basis to inform an integrated service response [21]. We frame health risk in the context of health as interaction of biological, environmental, and lifestyle factors, unique to the individual that has the potential to result in negative consequences [22]. Failure to take a personalized approach has proven to be burdensome both socially and economically [23,24,25]. The health care system is a prime example, where failed or fragmented interventions are eroding financial sustainability and improvement in outcomes, particularly for populations with multiple chronic health and social conditions [4,26]. On the individual patient-level, fragmented care has been associated with wasted resources, medical errors, poor health outcomes, and dissatisfied patient experience [27].

In overview, the problem of multiple chronic conditions and their financial impact on the health care system is a worldwide concern, escalating unease for reduced cost/benefits [4,28,29,30,31,32,33]. Currently, there are few interventions that explicitly identify and appropriately consider the relevant set of important socio-cultural determinants that affect people and their health and link these together into meaningful actions, such as integrated case plans to inform services. There is even less attention given to how the patient’s clinical and social condition interacts in the context of intervention and models of care. The challenge has been in focusing on the patient-centred nature of interventions in addressing behavioural, emotional, situational, and cognitive barriers in addition to the medical condition; all within the larger service system and ensuring that client services are all working together in supporting optimal outcomes.

This article takes up the challenge by drawing on theoretic and methodological extensions of two prominent approaches; ecological systems theory and social network analysis. Considered in combination, these two seemingly disparate approaches suggest a powerful new way of thinking about person-centered approaches as well as offering a methodologically stronger and more rigorous set of analytical tools. The model developed from this combination offers to bridge the apparent disconnect between service integration levels and offers a way to measure and assess integrated care with client needs in such a way as to direct optimal effort to interventions at the individual level. This will provide a new approach to looking at integrated care and will address the gap in the literature of how design and assessment of integrated care might be developed and examined. Shifting the focus on relationships between and among providers, and measuring and mapping connections and flows between individuals, groups, and organizations will provide a new way of thinking in bridging the disconnect between service levels [34].

The ecological systems theory approach is here considered in terms of developmental social ecologies (DSEs), as elaborated by Bronfenbrenner [35,36,37]. The theory has been extended recently as an algorithmic computer-based analysis by Lipina and colleagues [38,39] and used extensively as the basis for implementation of service provision that overlays and integrates individual needs and interactions within four (and sometimes five) system levels. Although the exploration of DSEs shares considerable synergy with explorations of social-ecological systems [10,11,40], the DSEs are generally confined to elaborations in terms of social systems rather than systems that include physical factors, such as water (although see some ecological considerations as they relate to DSEs) [39].

The second approach, social network analysis (SNA), provides a way to link actions within and across the various service systems and individual personal layers [41,42,43]. SNA demonstrates via visual graphics and metrics the type and level of connection between risk factors and service needs, and in so doing, reduces the need for the complicated analysis previously required. As well, SNA offers theoretical insights garnered by viewing integration through a complexity conceptual lens [44].

As a combined product of these two approaches, this article introduces a multi-level logic model or framework that ‘connects the dots’, within and across different levels, in order to focus on individual outcomes based on community-based appraisal of risk that is person-centred and place-based. An additional function of this article is to suggest potential future research directions related to integration of service systems more purposefully with the needs of the client base, including recipients of health care as illustrated herein.

The article is arranged in four sections, with the first section outlining the ecological systems theory archetype and subsequent extensions. The second section further explicates ecological systems theory through an application of nested networks. The third section summarises how SNA has been used successfully in studies of client service systems, highlighting its analytical potential, including the crossing of multiple systems levels. The fourth section blends the nested network adaptation of ecological systems theory with the SNA approaches discussed in section three. This has enabled the development of a proposal for a service integration implementation system based in a ‘social ecology network’ approach, which can be examined both qualitatively and quantitatively in future research.

## Theory and Discussion

Various research approaches and theoretical stances have been used to understand, explain and develop interventions for complex service problems, including for example, strength-based models, joined-up services and collaborative practice [45,46,47]. Despite the insights offered, invariably these approaches and theories have focused attention narrowly on either the service system or the person as a user, overlooking the dynamic patterns of interaction and influence that take place between people and the broader systems of influence in which they are situated [48]. As a result, primary care has been overlooked in its potential in providing comprehensive care that integrates and coordinates the care of all patient health needs and engages individuals, families, and communities [49].

The proposed model is grounded in the theoretical framework of social network theory and its application [50,51]; social network theory “refers to the mechanisms and processes that interact with network structures to yield certain outcomes for individuals and groups.” [51, p. 1168]. Social network theory describes the structure and properties of interactional links between individuals, groups or organizations that comprise a social network [53]. Linking social network theory with ecological systems theory provides a framework for conceptualizing interactional links along multiple levels of relationships, extending social networks into community contexts.

### The ecological systems theory approach and developmental social ecologies (DSEs)

Ecological systems theory is a widely-cited socio-cultural approach introduced to overcome such artificial divisions, offering a more holistic perspective that acknowledges and captures the complex interplay between individual, relationship, community and societal factors, including physical or environmental factors. Together, or individually, such factors can either put people at risk or act as protectors, thus adding to the overall complexity of intervention systems and confounding intervention and integration efforts. In this approach, people’s situations and the way they are responded to are viewed not as general but as interwoven into the individual socio-cultural (and organizational) domains in which they reside [54].

Arising initially from the work of Bronfenbrenner [35,36] but adopted widely across a number of differing service sectors (health, education, legal), ecological systems theory has been recently introduced and applied to service systems more broadly. This socio-cultural view, originating in psychology, often discussed in terms of DSEs, presents a person’s life as comprised and shaped by four levels of influence, micro, meso, exo and macro systems, where these levels described individual, social, institutional and cultural or ideological environments, respectively. The dimensions and features of each are expanded below. The approach elaborated by Bronfenbrenner [35,36] overlays individual need, and the interactions within four levels of systems integration. Described as “nested arrangement of structures, each contained within the next” [35] these layers and related factors must be examined as an interdependent whole to fully understand the forces surrounding an individual. A locational aspect is also included by viewing each system as arising from a setting, defined as “a place where people can readily engage in face to face interaction” [35].

The **micro system level** contains those elements or factors that are closest, or proximal to the individual person at the focus of the ecology, the central person. These can be thought of as those elements or factors with which the individual interacts directly, and where these play a role in shaping the individual’s experiences though bi-directional interactions [35,36,37,55]. The setting here relates to where proximal interaction takes place for that central person. Intervention efforts at this micro level involve establishing a clear understanding not only of the central person’s needs, but also the proximal social and cultural connections directly relevant to their life. This approach also more explicitly directs intervention efforts toward inter-personal relationships and interactions between services and the persons they are there to assist.

The **meso systems level** comprises the linkages and processes taking place between two or more proximal elements or factors containing the central person (i.e., it is a system of micro-systems). The micro system level is nested within the meso system level, which may include social interactions between members of institutions and groups involved in the micro system. The meso system relates the individual to the ‘neighbourhood’ setting of culture and society, including family, peers, school, practices and socio-economic status of the community in which that person is situated. Intervention efforts at the meso level will be related to interactions between people who are responsible for the central person’s welfare and development, such as interaction of a family physician with multiple health care providers on the patient’s condition.

The **exo system level**, in which meso systems are nested, includes individuals and other environmental elements that influence the central person but in which they do not participate directly. The exo systems in this level, for example, comprise the interactions taking place between two or more individuals in the social settings of groups or institutions, at least one of which does not include the central individual. These meso level interactions, however, indirectly influence processes within the central person’s proximal system. In a health care setting this may be seen as a physician taking on administrative responsibilities, resulting in less time for patient consultation indirectly affecting the individual patient’s access to care.

The outer **macro systems level**, in which exo systems are nested, includes broad cultural influences and ideologies that may have indirect, but long-ranging consequences for the central person. The macro system consists of an overarching setting that embraces micro, meso and exo systems. For example, cultural contexts and societal values embedded as educational systems, legislation, regulatory, or policy making actions, comprise exo system elements that have the potential to shape intervention programs and practices, as well as more generally influence the life of a central person indirectly through the other system levels. Intervention at this level may require system changes such as a change of government policy that leads to a change in the circumstances of the central person.

An additional **chrono system** adds a temporal dimension to studies that are examining the other four systems and their interactions over intervals of a given time period [38]. How a central person is situated within the ecological system today needs to be related to past events. Undoubtedly, this necessarily includes a historical view of system change and includes patterning of events, transitions and socio-history over a person’s lifetime.

This nested systems level approach serves to highlight the interplay between temporal and developmental as well as place-based and context-based socio-ecological factors and their service connections, providing detailed insights into system modulators and in reducing negative effects of uni-disciplinary perspectives on interventions. The use of DSEs has helped to better understand systems complexity and the challenges of intervention, including pointing to the gaps and points of disconnect. Effectively applying DSEs allows different practitioners to work in an interdisciplinary way towards intervention by reducing the impact of their own uni-disciplinary perspective [38]. In order to move forward, a mechanism is required that has the capacity to function in visualising interactions within and across all levels.

### Nested levels to nested networks

The nested systems conceptualisation articulated here has proven useful in understanding connections and their flow-on impact and has highlighted more dynamic conceptualisations that are person-based. However, more recent theorists have pointed to the limitations of Bronfenbrenner’s linear conceptualisations of social systems as encapsulated by the nested circles, arguing that they are more realistically viewed as overlapping arrangements of structures, each directly or indirectly connected.

**Figure 1:**
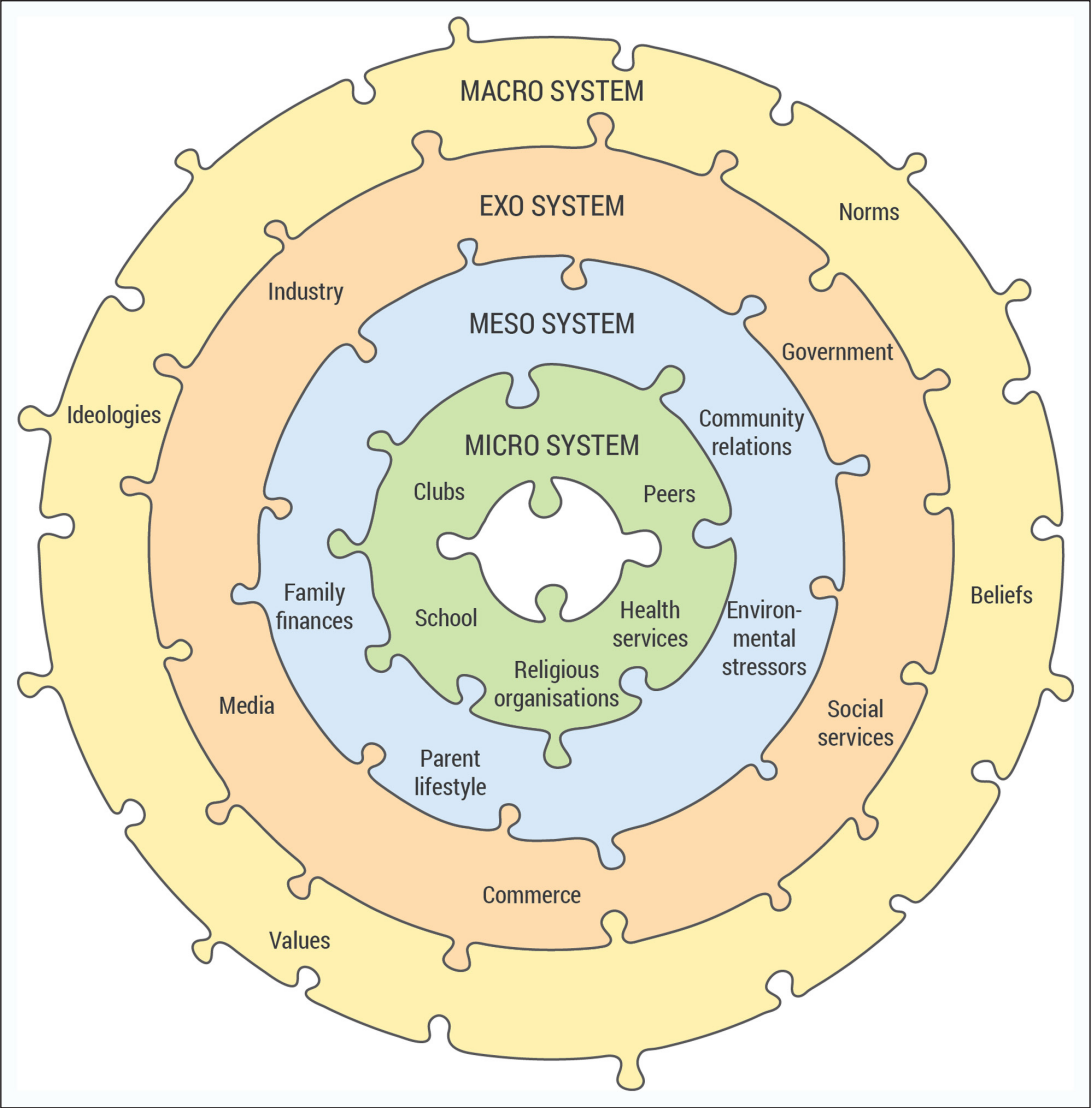
A diagrammatic representation of a simple social ecology framework, adapted from Bronfenbrenner [35,36,37].

In a recent theoretical study Neal and Neal [56] responded by extending the conceptualisation, constructing a nested network model that views ecological systems as overlapping arrangement of structures, each directly or indirectly connected. This network adaptation arises from the application of SNA to considerations of DSEs in order to allow a structural picture to emerge of the interrelationships of individuals and factors. In this conceptualisation, exemplified in an educational context, a school child’s relationship to the principal, teacher and coach is considered as a school microsystem, and the child’s relationship to father, mother and sibling is considered as the family microsystem. The interaction of these two systems comprises the meso system and in this way the description varies from the more linear system in Figure 1, where all actors except the child would be in the micro system.

Although Neal and Neal’s [56] approach has contributed to a reconceptualization of the nature of the connections and has forecast the methodological capability of multilevel approaches of DSEs, it has not been extended to empirical studies. Nonetheless, it remains a valuable descriptive model offering a novel view of ecological systems theory and, for us, at least offered a valuable clue to ways forward through a link between a well-known methodology used for examining service integration and the needs-based statistical analyses provided by a Diversified Derivation Algorithm (DDA) [38]. DDA takes into account a combination of varying individual and contextual links, belonging to several levels of analysis, and informing action on specific combinations. As a result, needed interventions can be identified at the individual and/or context level (i.e., family, community etc).

The argument presented here is that, so far, the approach to delivering services has mostly been conceived and implemented at single levels yet, as indicated above, people function within multiple levels of relationships, which are themselves variously connected. The networked theory of DSEs, therefore, should likewise be multi-level in its approach and scope, and offer services at multiple levels. Conversely, individual cognitions, attitudes, and behaviours for example can also influence the functioning and outcomes of teams and organization at different system levels.

### Connecting people and services using social network analysis: A multi-layered and multi-dimensional approach

The missing link between multi-level and social network perspectives is interactional on systems of nested networks [57] which suggest that each node in a network at a given level of analysis is itself a network at a lower level of analysis. Leveraging the idea of nested networks, multi-level and social network theoretic perspectives have been coupled to show how an observed network structure at one level of a system relates to and informs intervention efforts at higher levels of analysis or operation. Social networks, with their ability to (a) create a localised (bottom-up and lateralised) client centric model and (b) to bridge levels of operation and analysis at the client level, are a way forward in investigating service integration as a complex system. SNA has proven useful in examining such networks with an objective to facilitate the construction and evaluation of fully integrated service delivery based in both individual and community needs [58].

SNA is a powerful and well-tested tool for representing and examining relationships in terms of system connectivity and follows well-established analytical methods that allow qualitative mapping and quantitative analysis of the edges and nodes [50]. In the social ecologies illustrated above, this would correspond to connections (edges) between people (nodes), although some nodes may be institutions or artefacts (such as a computer login). Social network maps offer a dynamic snapshot of interactions across system categories and enable diagnosis and evaluation for use in planning of intervention and support [59]. Their advantage lies in the use of sophisticated software, such as UCINET [60] that enables fast analysis of big data sets, as well as supporting network metrics.

Use of SNA has provided a way to integrate within and across the service and systems as well as both horizontal and vertical individual networks [61,62]. As well, SNA demonstrates via visual graphics and metrics the type and level of connection between risk factors and service needs. In this context, SNA presents as a unique bridging and visualising mechanism, showing the links between factors, and where the points of intervention are best implemented. Importantly, rather than with specifics of the features and interactions or the attributes of individual actors within the network, SNA concerns itself with understanding how the features of a complex system arise from their underlying network structures [53].

### Pulling it all together: The ‘social ecology network’ model

**Figure 2:**
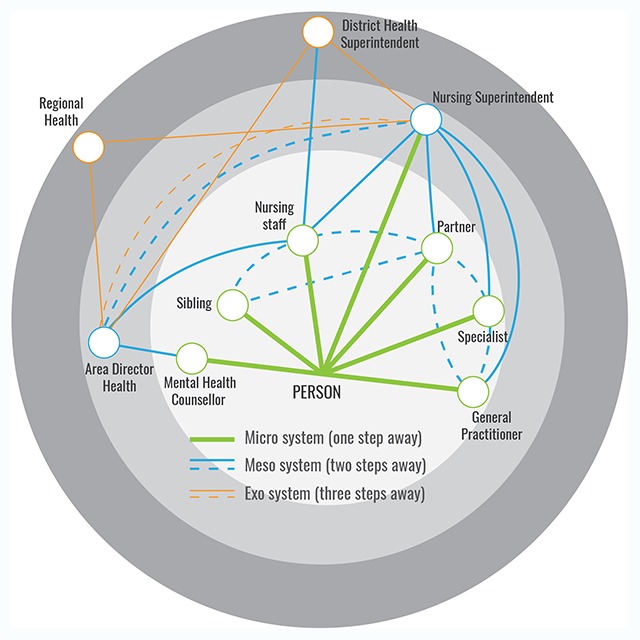
The social ecology network, with micro system connections in green, meso blue and exo orange (Copyright Woolcott, used with permission).

The logic of the layered interaction of ecological systems theory and its network adaptation led us to combine these using SNA to create a ‘social ecology network’ model. The model, illustrated in Figure 2, is based in the factors or elements connected to the central person and mapping of the pathways to factors from that person outward, including support services and potential risk or protective factors. This makes the entire network person-centred and allows a pathway analysis that refines the average path length data from SNA, providing a more nuanced interpretation of the connection of the central person across systems rather than system levels. As shown in Figure 2 (green edges) the micro system comprises the ego net of proximal factors directly connected to the central individual. This includes all connections that are one step away from the central individual and related directly to a person’s lived experience or context, including social factors that may be considered as needs or risk factors.

**Exhibit 1: A vignette outlining the need for an already connected system of service provision for an individual**

This vignette demonstrates the need for the social ecology model outlined in this article as a basis for an integrative care framework that connects the multiple needs of an individual in an efficient and effective way (i.e., smaller path lengths and less pathways from individual to service provider). The vignette draws from a place-based SNA study of homelessness services systems in Queensland Australia [45,46] and illustrates the complications in service provision that can ensue if there is no overall framework, in this case necessitating the superimposition of an individual’s own calculation of his ego-net over a network of organisational service provision, each for multiple individuals.

Mr X presented to a homeless service provider seeking assistance in securing accommodation. Like many people experiencing homelessness Mr X also suffered from multiple health and social issues. He reported that previous efforts to source help have been largely an unsuccessful and unsatisfactory experience. While Mr X acknowledged that he has multiple support needs, he indicated that only rarely has time been taken to genuinely explore and understand his needs or ask what he wanted from the service system. Frustrating Mr X also was the tendency of services to take charge of his ‘care’ (having identified permanent housing as a first step) overlooking his capacity to make informed decisions based on his experiences and knowledge of his situation and ability to draw on other supports. Furthermore, Mr X’s search for accommodation and other support was hampered by the array of services available, each with differing service offering and eligibility requirements, and the lack of integration between these services.

Mr X’s perception of fragmentation was confirmed by two SNA studies which found the system to be quite sparsely connected owing to two competing service hubs, requiring on average 2.4 steps (visits) before Mr X received support [65,66]. The lack of a ‘one stop shop’ option, and apparent disconnections between services, obligated Mr X to personally search out the services and present to several, repeating his testimony or story. Understandably, on presenting to the agency worker, Mr X was annoyed and frustrated at the number of places he had to attend and services at which he had to repeat his story before being referred to the ‘right door’.

Understanding his frustration and appreciating MR X’s right to and capacity for self-management, the service provider set out to provide him with the knowledge to navigate the system more efficiently in the future. This was achieved through three steps.

1. Using the SNA maps (which examined homelessness and related services) the composition of the local service system and the connections between them was identified at multiple organisational levels (i.e., local, state, national). It was envisaged that having a working understanding of the service system, including location and purpose (eligibility and referral criteria) provided a level of transparency that wasn’t present earlier, thus enabling Mr X to have a clearer picture of the service system, how it was connected and therefore operated, including gaps and overlaps.
2. The organisational (system) levels having been mapped and unpacked, Mr X was then tasked with drawing a personal socio-gram (or ego-net in SNA terminology) identifying his personal and service support network; the different groups of people and organisations with whom he interacted and the nature or relationship of these interactions (e.g., positive or negative). This enabled Mr X to understand the strengths and weaknesses of his networks, helping his capacity to manage these, and know where to intervene or what to change to support his ‘care’.
3. Finally, Mr X’s personal network socio-gram was superimposed onto the wider service system network to demonstrate how his network might fit into the wider service network. Based on this enhanced knowledge it was expected that Mr X would be better able to navigate the services in the future.

Based on the ecological systems models described in previous sections, connections of these micro system factors to non-proximal factors that are one step further away effectively comprise the meso system—each factor is two steps away along a connection pathway from the central person. A nursing staff, for example, in discussing a person (the patient) with his/her partner or sibling is not directly relating to the person and this interaction is in the meso system (dotted blue line in Figure 2). This differs from the model of Neal and Neal (2013), [56] where the principal/coach interaction (corresponding to the Nursing staff/partner interaction in Figure 2) is included in the school micro system (although again as a system rather than a level).

The use of a network connectivity model here, suggests that there is also a second set of connections, not confined strictly to the micro system level of the ecological systems models that are one step away (proximal) from the central person and therefore part of the micro system of that person. For example, a patient may have an occasional direct connection with a nursing superintendent (the longer green line in Figure 2), rather than a connection through a nursing staff as an intermediary. This would make the nursing superintendent a micro connection to the patient (one step away) as well as a meso connection through the partner and nursing staff (two steps away).

Additionally, connections between proximal factors (the dotted blue lines in Figure 2) may be considered as part of the meso system, since some or all of these factors may be two steps away from the patient. A connection between the partner and the specialist, for example, may be a meso system connection that does not directly involve the patient. This may conflict in some ways with a connection strategy, common in SNA, of removing the ego from a diagram and connecting the ego factors as a network. The assumption of meso for proximal elements in such a diagram, therefore, may need to be validated by proof of an actual meso connection (i.e., determining whether the partner is in reality connecting with the specialist).

Connectivity of the network can be continued outward from the central person in a similar way to form the outer exo, macro systems and potentially mega systems. As the pathway out from the central individual is considered, for example, single pathway connections from or between meso system factors may be considered in this model as comprising exo systems. These connections would be, in effect, three steps away from the central person. For example, a district health superintendent who interacts with both the nursing superintendent and the partner is acting from the exo system, provided that the nursing superintendent is acting through, say, the partner (in the meso system). The nursing superintendent’s interaction with the patient may also be considered as exo, since this may be three steps away from the patient if it is through the district health superintendent and then the nursing staff.

A major difference between this model and previous social ecology conceptualisations is that connections in this social network approach can be mapped across more than one level, meaning that any counter-productive limitations of previous models pertaining to interaction between levels, or its lack, may be reduced. This dual/multiple connectivity of the social ecology network, for example, offers potential for testing of hypotheses related to how the different system connectivity’s from a central person might correlate with service delivery optimisation or intervention success (see vignette in **Exhibit 1**). Previous network analysis, for example, has shown that service interventions that target a particular person are optimized if average path length (from person to services) is low [58,63,64]. This suggests that the service provision based on need must be a small number of steps away from, and both directly and indirectly connected to the person, including through risk factors or protective factors.

An additional advantage of the social ecology model is that SNA allows for consensus networks to be constructed that would allow comparison of a single person’s social ecology network with multiple and complex needs with that of other persons combined in such a consensus. This model, therefore, allows a view of service provision that embeds it in a person-centred network where consideration of the proximal factors that are associated with risk are examined in conjunction with consensus networks that consider a larger person population and their common system factors, from micro outwards.

Furthermore, the model allows for analysis by way of testing a hypothesis that pathways from support service providers to each supported client are more effective and efficient if they provide the shortest path to, or least number of intervention steps, compared with the model. For example, SNA could be used to examine nodal sequences that had proven significance (through statistical analysis) in providing effective client support from various services, such as outlined in the vignette in **Exhibit 1**. If consensus is used, the SNA metric of *betweenness* could be used to weight nodes through the number of clients who engaged with the effective services (the service pathway in this model). *Betweenness* is related to the number of connections between two nodes, in this case the clients and the services they are accessing, and measures how important the node is in traversing the network.

This style of multifactorial analysis, if based in localised contexts should, therefore, render efficiencies in service provision through examining whether the needs of individuals across a selected community are being met within available constraints, and whether these constraints are related to fiscal, social, organisational or other factors in the various outer systems. It may well be, for example, that multiple pathways of one to three steps are required from meso levels to the central person, but that multiple pathways of more than three steps to outer systems are inefficient, depending on context. Effectively, the model can be framed as an extended personal community [70], that is, a community of actors around an individual, as understood from the perspective of that individual.

These social ecology networks, which are of their very nature place-based, comprise a broad set of interrelated factors at multiple levels of interaction and are connected in complex ways, in a similar way to social-ecological networks, such that they embrace a complex systems perspective [11]. The solution to problems with effective service provision, therefore, is to locate these services in such ecologies in order to improve interventions and programs through appropriately targeting need. In other words, the use of the person-centred and place-based model may be more effective because it pays attention to the needs of each person, given that they may each be in a particular place. Adaptation of the model means that a tool can now be developed to improve the social ecology networks of persons who may be at risk of poor or ineffective service provision, based on comparison of their network with consensus networks of persons who are not at risk, with these being idealised as success networks [67]. Additionally, these same networks could potentially be viewed in real time at different intervals, giving a continuing representation of risk across a cohort or service sector.

## Conclusion

In conclusion, we must fundamentally rethink the way we address complex health and social problems. The focus requires a shift from specific diseases or conditions to the individual needs as not everyone that has disease, has it for the same reason(s) or experiences it the same way. Emphasis should be put on the larger context and on the interrelationships which shape social and environmental determinants of health. Furthermore, interventions that target social and health problems, such as violence, poverty, addiction, depression, neglect, homelessness, and elevated school dropout rates, depend on cooperative social connections based on interdependence and reciprocity of interrelated communities of practice [68].

Multi-level network studies considering interdependencies that exist between multi-level networks are infrequent in the literature, though some studies investigate relationships between micro-level and macro-level networks in the context of organizations [61,69,42]. Nevertheless, many questions remain unanswered in advancing knowledge of complex-cross-level processes leading to the emergence of patterns of relating in supporting a person-centred and place-based model of care. Research that analyses and explains emerging patterns of relationships at different levels in social ecology networks and uses them to explain outcomes is clearly needed. This article provides a conceptual framework for moving forward in this respect.

Growing evidence supports the need to turn intervention to more person-centred approaches that configure context according to place. These approaches must incorporate complex sets of interaction of socio-cultural elements related in an ecology and, in doing so, must also highlight and distil risk along with protective factors so that all ecological determinants are explicitly identified and incorporated into the design and implementation of interventions. Leveraging the idea of nested networks, the model outlined here links multi-level and social network theoretic perspectives to show how an observed network structure constructed from a person centre through direct contact, or proximal, factors in that person’s socio-cultural context, relates to and informs intervention efforts at higher levels of analysis and/or operation.

This approach has particular relevance for people with multiple and complex needs, since current approaches are known to be failing in service provision and are proving extremely costly to maintain. Shifting the emphasis and extending the network array across micro, meso, exo and macro systems brings a new way of thinking, acting and organising practice. This acts on the realization that people operate within systems to provide service but drills down to the individual level obtaining ownership that relates to appropriate provision of that service.

Future person-centred and place-based studies may do well to collect a broad and complete set of data across demographics, including survey data as well as archived data, so that risk factor networks may be more closely tied to particular social ecologies. Such an approach can prove to be very useful in addressing future research questions, such as: How might social systems incorporate mental health needs of vulnerable populations?; and, How can different social and environmental dynamics be linked to minimize individual health risk and optimize health outcomes? Our proposed model offers a new way of thinking about person-centered care, as well as offering a methodological approach to advance research and practice.
